# Characterization of pPCP1 Plasmids in *Yersinia pestis* Strains Isolated from the Former Soviet Union

**DOI:** 10.1155/2010/760819

**Published:** 2010-12-20

**Authors:** Chythanya Rajanna, Tamara Revazishvili, Mohammed H. Rashid, Svetlana Chubinidze, Lela Bakanidze, Shota Tsanava, Paata Imnadze, Kimberly A. Bishop-Lilly, Shanmuga Sozhamannan, Henry S. Gibbons, J. Glenn Morris, Alexander Sulakvelidze

**Affiliations:** ^1^Emerging Pathogens Institute and Department of Molecular Genetics and Microbiology, University of Florida College of Medicine, Gainesville, FL 32610, USA; ^2^National Center for Disease Control and Public Health, Georgian Ministry of Health, 0177 Tbilisi, Georgia; ^3^Tbilisi State University, 0218 Tbilisi, Georgia; ^4^Naval Medical Center Annex, Rockville, MD 20852, USA; ^5^US Army Edgewood Chemical Biological Center, Aberdeen Proving Ground, MD 21010, USA

## Abstract

Complete sequences of 9.5-kb pPCP1 plasmids in three *Yersinia pestis* strains from the former Soviet Union (FSU) were determined and compared with those of pPCP1 plasmids in three well-characterized, non-FSU *Y. pestis* strains (KIM, CO92, and 91001). Two of the FSU plasmids were from strains C2614 and C2944, isolated from plague foci in Russia, and one plasmid was from strain C790 from Kyrgyzstan. Sequence analyses identified four sequence types among the six plasmids. The pPCP1 plasmids in the FSU strains were most genetically related to the pPCP1 plasmid in the KIM strain and least related to the pPCP1 plasmid in *Y. pestis* 91001. The FSU strains generally had larger pPCP1 plasmid copy numbers compared to strain CO92. Expression of the plasmid's *pla* gene was significantly (*P* ≤ .05) higher in strain C2944 than in strain CO92. Given *pla*'s role in *Y. pestis* virulence, this difference may have important implications for the strain's virulence.

## 1. Introduction

*Yersinia pestis*, the causative agent of Black Death, is a highly virulent bacterium responsible for an estimated 200 million human deaths throughout recorded history. The bacterium is believed to have evolved from the much less virulent *Y. pseudotuberculosis* relatively recently on the evolutionary scale, approximately 1,500–20,000 years ago [1]. In most of the developed world, the genetic organization, virulence mechanisms, and life cycle of *Y. pestis *have been extensively studied using a few *Y. pestis *strains [2], some of whose genomes have been fully sequenced [3, 4]. In contrast, there remains a striking paucity of data concerning the genetic organization, virulence traits, prevalence, and epidemiology of *Y. pestis *in other parts of the world, including the Republic of Georgia and other republics of the former Soviet Union (FSU) [5–7]. 

The evolution of *Y. pestis*, and much of its virulence, is due to its acquisition of plasmids. Three plasmids (pCD1, pFra, and pPCP1) are typically present in all biovars of *Y. pestis* although additional plasmids, many of which are cryptic, have been reported [8–11] to be present in several worldwide strains of *Y. pestis*. pCD1 (also designated pCad, pLcr, pVW, and pYV) is a 68 to 75 kb plasmid found in all three currently recognized pathogenic species of *Yersinia*: *Y. pestis, Y. pseudotuberculosis,* and* Y. enterocolitica* [9, 12]. It contains genes encoding several essential virulence determinants, including the highly conserved low-calcium response stimulus protein (LCRS), which has both regulatory and antihost functions and the *Yersinia* outer membrane proteins (Yops).

The pFra plasmid (also designated pYT and pMT1) is unique to *Y. pestis.* It is typically ca. 100-kb, but its size can vary drastically among various strains, ranging from 60 kb in the deleted version of the Dodson strain [13] to 280-kb in other strains [14]. The plasmid's role in *Y. pestis* virulence is not fully understood, but it is known to contain genes that encode two putative virulence factors: (i) the F1 protective antigen associated with increased resistance to phagocytosis by monocytes and (ii) the *Y. pestis* murine toxin (YMT), a phospholipase D encoded by *ymt*, whose intracellular activity protects *Y. pestis* from digestion in the flea gut; thus, facilitating the bacterium's ability to colonize the flea's midgut and to increase its arthropod-borne transmission [15]. 

pPCP1 (also designated pPla, pYP, and pPst) is another plasmid unique to *Y. pestis* and has a size of approximately 9.6 kb. In addition to possessing a few regulatory genes and genes encoding “hypothetical proteins,” it contains *pla*, *pst*, and *pim*, which encode a plasminogen activator (PLA) protease, the bacteriocin pesticin, and a pesticin immunity protein, respectively [9]. Its size and genetic organization are usually similar among various strains of *Y. pestis* although some isolates of the bacterium may lack the plasmid or may contain a multimer of the plasmid [8, 14, 16]. pPCP1 is an important virulence determinant in *Y. pestis* because it contains *pla*, the gene that encodes the PLA protease virulence factor. However, despite the importance of pPCP1 and other plasmids for *Y. pestis* virulence, there is a lack of information about the genetic structure of the plasmids in FSU strains. During the studies reported in this communication, we (i) determined the complete sequences of pPCP1 plasmids in three *Y. pestis* strains isolated from the FSU and examined in a recent study [7], (ii) compared their nucleotide sequences with those of pPCP1 plasmids in three well-characterized, non-FSU strains, and (iii) determined the relative pPCP1 plasmid copy number of three FSU *Y. pestis* strains.

## 2. Materials and Methods

### 2.1. Bacterial Strains

pPCP1 plasmids were extracted from bacteria in a recentlycharacterized FSU *Y. pestis* strain collection [7]. The pPCP1 plasmid from strain CO92 was used as a control. Multiple small specimens of each strain were stored at −80°C in 70% Luria-Bertani (LB) broth supplemented with 30% glycerol (v/v), and each aliquot was used only once before being discarded.

### 2.2. Multilocus Variable Number Tandem Repeat (MLVA) Analysis

Our MLVA analysis used a simple 7-polymorphic marker (ms01, ms04, ms06, ms07, ms46, ms62, and ms70) protocol previously described by Pourcel et al. 2004 [17]. The primers used for PCR amplification are listed in  Table  1 (supplementary material). The PCR reactions were performed with aliquots of purified chromosomal DNA (50 *μ*L containing 1 to 2 ng) and Choice-Taq Blue DNA polymerase (Denville Scientific, Metuchen, NJ). The sequential reaction conditions were (i) 96°C for 5 minutes, (ii) 34 cycles of denaturation (96°C, 20 seconds), (iii) annealing (54°C, 30 seconds), (iv) elongation (72°C, 1 minute), and (v) a final extension step for 5 minutes at 65°C. The PCR products were purified with a Qia Quick 96-well Plate Kit (QIAGEN, Valencia, CA), and the purified PCR products were sequenced in both directions with a BigDye 200 Terminator Cycle Sequencing Kit and a 201 ABI 3730xl 202 DNA analyzer (Applied Biosystems, Foster City, CA). The individual sequences were analyzed with a Tandem Repeat Finder (TRF) program (http://tandem.bu.edu/trf/trf.html) and the number of repeats for each marker was determined.

### 2.3. PCR Amplification of the pla Gene and Plasmid Extraction

Well-isolated colonies of each strain were obtained by streaking and incubating (28°C) the bacteria on brain heart infusion (BHI) agar. After 48 hours, 5 mL aliquots of BHI broth were inoculated with well-isolated colonies of the strains and incubated at 28°C for 24 hours with agitation (200 rpm). DNA was extracted from the bacteria in 3 mL aliquots from each broth culture, and the *pla, pim, *and *pst* genes were PCR-amplified using primers plaF1, plaR1, pimF, pimR pstF, and pstR (Supplementary Material  Table  1 available at doi:10.1155/2010/760819), which amplified 470-, 201-, and 197-bp regions of the respective genes. The cultures which were PCR-positive for all three genes were streaked on BHI agar and incubated at 28°C for 24 hours. Aliquots of 100 mL BHI were inoculated with one loopful of culture from the streak plates. After incubation with agitation (200 rpm) at 28°C for 24 hours the bacteria were collected by centrifugation, and plasmid DNA was extracted with a QIA filter Plasmid Midi Kit (QIAGEN) according to the manufacturer's instructions, and the DNA specimens were characterized by agarose gel (1%, w/v) electrophoresis. The reproducibility of the data was confirmed by repeating the experiments at least twice for each strain.

### 2.4. Sequence Comparison and Confirmation pMT1-pCD1 Chimera Plasmid

To determine the nature of atypical plasmid seen in C790, we obtained 454 sequence of the plasmid and genomic DNA and compared this sequence to the respective plasmids of CO92 genome (Sozhamannan laboratory, unpublished results). To confirm the atypical plasmid  as  a  chimera of pMT1 and pCD1, PCR primers sulk560  (ACTCACGCAGCGTATCTTCC, pMT1) and sulk561  (ATTCTCTGTCGTTCGGCTTG, pCD1) were designed to amplify across the junction by PCR.

### 2.5. Sequencing the pPCP1 Plasmids

The nucleotide sequences of the extracted pPCP1 plasmids were determined by the primer walking approach. The primers used for sequencing are listed in  Table  1 (Supplementary Material). The plasmids were sequenced, in both directions, with a BigDye 200 Terminator Cycle Sequencing Kit and a 201 ABI 3730xl 202 DNA analyzer (Applied Biosystems). Contigs were assembled with Phred [18] and Phrap (available at http://www.washington.edu/) programs. The contigs were viewed with the Consed program [19], and the resulting DNA sequences were trimmed by removing low-quality nucleotide sequences from the end. The sequences were aligned by the Sequencher 4.7 program. The final sequences were compared with those of previously characterized pPCP1 plasmids in three non-FSU *Y. pestis* strains (KIM, CO92, and 91001). The latter sequences were available from the Institute of Genomic Research (TIGR) database  (http://www.tigr.org/). The genetic relatedness among the plasmids was determined by the neighbor joining tree method, using the BLOSUM62 of EMBL-EBI ClustalW2 program (http://www.ebi.ac.uk/).

### 2.6. Quantitative PCR of DNA and RNA

For extracting genomic DNA and total RNA, cultures were grown over night in 5 ml BHI broth at 28°C shaking at 200 rpm after inoculating fresh well isolated colony from a BHI agar plate. For total RNA extraction 5 ml of fresh BHI media was inoculated with 500 *μ*l of overnight culture and incubated shaking (200 rpm) at 28°C for 4 h. 

Total DNA was extracted with a Genomic DNA Purification Kit (Promega, Madison, WI), and the DNA concentration in the extracts was adjusted to 100 ng/*μ*L. Primers for the *pla* gene (plaF2 and plaR2, Supplementary Material  Table  1) were used to estimate the copy number of the pPCP1 plasmid, and glnF and glnR primers (Supplementary Material  Table  1) for the chromosomal, single-copy *glnA* gene were used as the control. A standard curve (for concentrations ranging from 10^4^ copies/*μ*L to 10^11^copies/*μ*L) was constructed in order to estimate the copy number of *pla*. An iQ Syber Green Kit (BioRad, Hercules, CA) was used for real-time PCR (RT-PCR), and the ratio of the *pla* starting quantity to *glnA* starting quantity was calculated and compared among the strains. The experiment was repeated two times and the average values were estimated.

Total RNA was extracted with an RNEasy Mini Kit (QIAGEN), and contaminating DNA was removed by passing the extracts through a gel matrix containing bound RNase-free DNase **(**QIAGEN). The RNA concentration in the extracts was adjusted to approximately 50 ng/*μ*L, and 100 ng aliquots in 25 *μ*L reaction mixtures were reverse-transcribed with an iScript cDNA Synthesis Kit (BioRad). RT-PCR was performed with reaction mixtures (25 *μ*L) containing a template composed of 2 *μ*L of the cDNA preparations. The relative expression of *pla* to the expression of the reference gene (*glnA*) was determined using the 2^−ΔΔC^_T_ method [20]. Statistical analyses of each set of the *pla *expression data were performed separately, with the GraphPad InStat (version 3.05) program (GraphPad Software, San Diego, CA). An unpaired *t*-test was used to determine whether the differences observed in *pla* expression among the four strains analyzed (C790, C2614, C2944, and CO92) were statistically significant. A *P*-value <.05 indicated a statistically significant difference between the results.

### 2.7. Nucleotide Sequence Accession Numbers

Complete sequences of the three pCP1 plasmids described in this study (extracted from FSU strains C790, C2944, and C2614) have been deposited in GenBank, under accession numbers BankIt1374063 C790 HM807366, BankIt1374063 C2614 HM807367, BankIt1374063 C2944 HM807368, and the partial sequence of C790 chimera plasmid of pMT1 and pCD1 BankIt1408393 Chimera HQ612242.

## 3. Results and Discussion

### 3.1. Genetic Relatedness of FSU *Y. pestis* Strains

**Figure 1:**
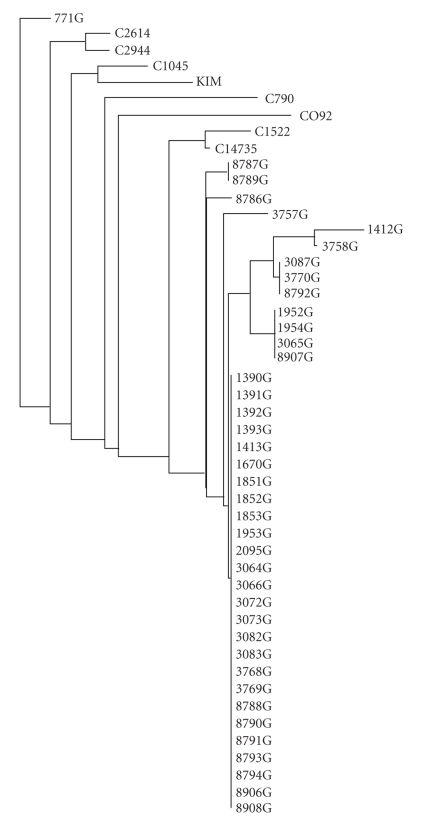
Neighbor-joining tree generated from MLVA data based on 7 loci.

A recent publication [7] describing the forty-six FSU *Y. pestis* strains isolated from the Republic of Georgia and surrounding countries used biochemical profiling, pulsed field gel electrophoresis (PFGE), and multilocus sequence typing (MLST) to determine the genetic relationships between Georgian *Y. pestis* strains and *Y. pestis* strains from neighboring countries and other parts of the world. It was found that the Georgian *Y. pestis* strains were of clonal origin and that PFGE discriminated the *Y. pestis* strains better than did MLST. In the present study, we expanded this investigation by analyzing the same strain collection with MLVA, which has been reported to be well-suited for differentiating various bacterial pathogens, including *Y. pestis* [17, 21, 22]. The results of the overall MLVA analysis were in agreement with those of the PFGE and MLST analyses: MLVA grouped the Georgian strains in three major clusters (Figure 1 and Supplementary Material  Table  2), except for strain 771G which was a clear outlier; this strain also clustered differently during previous studies [7]. Further, in agreement with the reports mentioned above, we found that MLVA differentiated the strains with greater sensitivity than PFGE and MLST. Specifically, MLVA was able to discriminate between Georgian *Y. pestis* strains that were grouped in a single cluster by MLST and PFGE in the previous study by [7]; for example, *Y. pestis* strains 8787G, 3757G, and 1412G were unresolved by PFGE typing, but they were differentiated by MLVA (Figure 1), which suggests that MLVA is better suited for specific identification of FSU *Y. pestis* strains than PFGE or MLST.

### 3.2. Prevalence of the pPCP1 Plasmid in FSU Strains

Some of the *Y. pestis* strains isolated from the FSU, including those isolated in the Transcaucasian and Daghestan Mountains; that is, two natural foci of plague adjacent to the areas from which our *Y. pestis* strains were isolated [7], have been reported not to contain the pPCP1 plasmid [14]. Many, but not all of these strains, are thought to belong to the so-called Pestoides biovar of rhamnose-fermenting *Y. pestis *strains commonly found in enzootic hosts, but not usually associated with human infections. Also, *Yersinia* plasmids are unstable and may be easily lost during prolonged storage and passaging. The *Y. pestis* strains in our collection were isolated during 1966–1997 [7], and they were regularly passaged during their storage, which could have further facilitated plasmid loss. Therefore, we first screened the FSU strains of *Y. pestis* by pPCP1-specific PCR (using the primers listed in Supplementary Material  Table  1), in order to identify the strains in which the plasmid was still present. Only three of the forty-six FSU strains we examined (C2614, C2944, and C790) gave strong specific positive PCR signals for all the three genes (*pla, pim,* and *pst*) known to be contained in the pPCP1 plasmid. Also, the buffer control and *Y. enterocolitica* strain ATCC 9610 (both negative controls) did not yield the PCR amplicons. The results of the pPCP1-targeted PCR screening study suggested that many of the FSU strains in our collection do not contain the pPCP1 plasmid, an observation that was further verified by direct plasmid extraction and other approaches described below. Even though in our previous report [7] we did report all the strains isolated from Republic of Georgia to posses *pla *gene, in this study, we find only 3 strains to posses the plasmid. We are still investigating the possible cause for this discrepancy and certainly will address in our future publications. Some possible reasons why this could have happened are 1. different batches of strains were used in each of this study, and there could be difference in plasmid composition of strains depending how they were handeled and shipped to us 2. there could have been low level contamination with a pla positive strain/ DNA in our first batch of strains.

### 3.3. Plasmid Extraction and Copy Numbers of pPCP1

**Figure 2:**
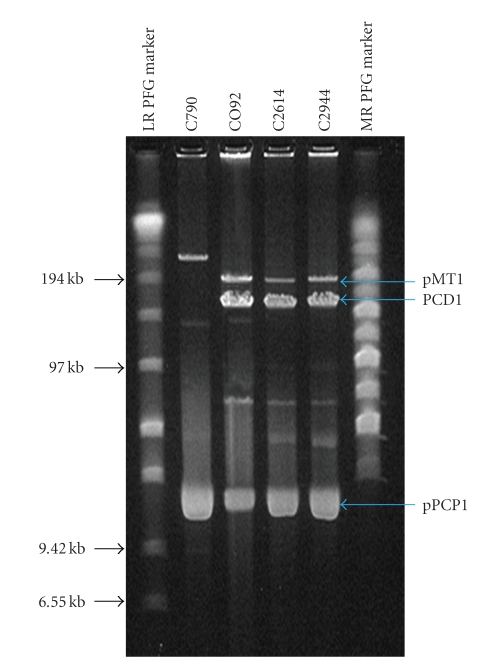
Pulsed field gel electrophoresis of total plasmid content on 1% agarose gel. Lane 1: low range PFG marker, lane 2: C790, lane 3: CO92, lane 4: C2614, lane 5: C2944 and lane 6: medium range PFG marker.

Efforts were made to extract the pPCP1 plasmid from all of the forty-six FSU *Y. pestis* strains in the collection, including three strains that gave positive signals for *pla, pim,* and *pst.* The pPCP1 containing, non-FSU strain CO92 was used as the positive control during plasmid isolation. Plasmid DNA was obtained from all three PCR-positive strains, but not from the forty-three *Y. pestis* strains that were *pla, pim, *and *pst *PCR negative, which supports our PCR screening data and indicates that PCR-based screening is useful for detecting the presence of the pPCP1 plasmid in *Y. pestis* strains. The presence of the pPCP1 plasmid appeared to be limited to the C2614-C2944 MLVA cluster and closely related clusters (Figure 1). It is possible that the strains in those clusters are inherently more stable in maintaining their plasmid composition; however, it is also possible that the strains in the most common cluster never contained that plasmid. Because of the known laboratory passage histories of these strains, resolution of this question may be resolved by ongoing efforts in the FSU to discover new epizootic strains. Moreover, the dearth of plasmid-positive strains does not allow for rigorous analysis of any possible association between plasmid-containing strains and their distribution among various MLVA groups or clusters. The plasmid yield from the three PCR-positive FSU strains and from CO92 that varied even though the strains were grown under identical conditions and their concentrations (CFU/mL) prior to plasmid extraction were about the same: approximately 250 to 300 *μ*g of plasmid DNA was consistently obtained from *Y. pestis* strain C790 compared to around 350 to 450 *μ*g from strains C2614, C2944, and CO92. Interestingly, FSU strain C790 lacked the 70-kb pPCD1 plasmid and its pMT1 plasmid was larger than the typical 96-kb pPMT1 plasmid (Figure 2). When 454 whole-genome sequence (WGS) output of C790 was compared to CO92 plasmid sequences, the alignment indicated that the atypical plasmid is in fact a chimera of pMTI and pCD1 with one junction being at NC_003131.1, position 65224 and NC_003134.1, position 73221. Individual sequencing reads that spanned this junction were clearly evident, and this conclusion was verified by PCR amplification of the expected 659 pb PCR product in C790 and but not in CO92 (Figure 4). We also notice a faint band in strain C2944 suggesting it may also have a chimera plasmid in low abundance along with typical pCD1 and pMT1 plasmids. The idea of the atypical plasmid being a chimera plasmid is supported by the fact that both *lcrV *(encoded on pCD1) and *caf1* genes were detected by PCR in this strain [7]. The second junction was not identified by WGS. Because of the tendency of bioinformatic assembly programs to collapse large repeats, we believe that the second junction is likely to lie in one of the large, highly repetitive IS100 elements that are found on all three plasmids and the chromosome. Identification of the second junction point, the full description of the chimera and its effect on pathogenesis will be the subject of future studies. Chimeric plasmids resulting from recombination between homologous sequences of *Y. pestis *plasmids have been documented; for example, the deep-rooted Angola strain contains a dimeric pPCP1 plasmid that is integrated at an IS100 element in tandem repeats into the pMT1 plasmid [23].

Quantitative PCR (qPCR) was used to verify the difference in the pPCP1 plasmid's copy numbers among the three FSU strains (C790, C2614, and C2944) and CO92, by comparing the *pla*/*glnA* gene ratios among them as there is only one copy of *glnA* in each cell [3]. The mean of three independent qPCR reactions (Table 1) showed that the highest copy number of *pla* in strain C2944, in which the *pla*/*glnA* ratio of 18.16 ± 2.99 suggested that there were approximately eighteen copies of the *pla* gene (and of the pPCP1 plasmid) in that strain. The next highest copy number was detected in *Y. pestis* strain C790, in which the *pla*/*glnA* ratio was 15.01 ± 1.14, followed by strain C2614 which had a ratio of 13.21 ± 0.64. Strain CO92, the virulent non-FSU strain that was used for comparison, had a *pla/glnA* ratio of only 6.48 ± 2.07. 

Although the absolute copy numbers of the pPCP1 plasmids are difficult to calculate based on the qPCR results (and the validity of the above qPCR-determined copy numbers must be interpreted with caution), the combined data suggest that the three PCR-positive FSU strains contain a larger number of the pPCP1 plasmid than does the non-FSU *Y. pestis* CO92 strain. Specifically, when compared to strain CO92, the FSU strains consistently had: (i) stronger PCR amplification signals and (ii) higher copy numbers when examined by qPCR. The underlying mechanism(s) for the difference in the copy numbers we observed is difficult to explain at the present time given the *rop *gene is identical in all the 4 strains. However, and as discussed below, it may have some important implications for the strains' relative virulence.

### 3.4. Genetic Organization of the FSU's pPCP1 Plasmids

**Figure 3:**
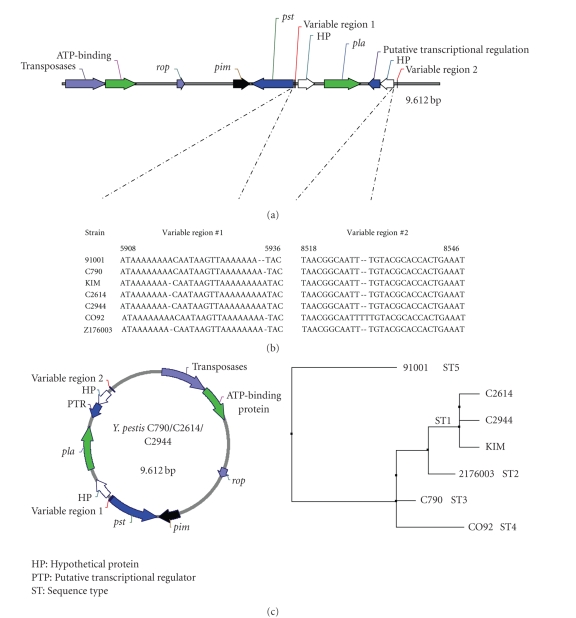
Structural comparison of the pPCP1 plasmids in the FSU's *Y. pestis* strains and a previously characterized strain *Y. pestis* CO92. (a) Schematic linear diagram of the pPCP1 plasmid in strain CO92, which shows the locations of variable regions no. 1 and no. 2 present in the FSU strains' pPCP1 plasmids. (b) Sequence variations in variable regions no. 1 and no. 2 in the pPCP1 plasmids examined during our studies. (c) Schematic composition of three pCP1 plasmids in the FSU's *Y. pestis* strains, and their genetic relatedness (based on neighbor joining tree analysis) to the three pCP1 plasmids from non-FSU strains.

**Figure 4:**
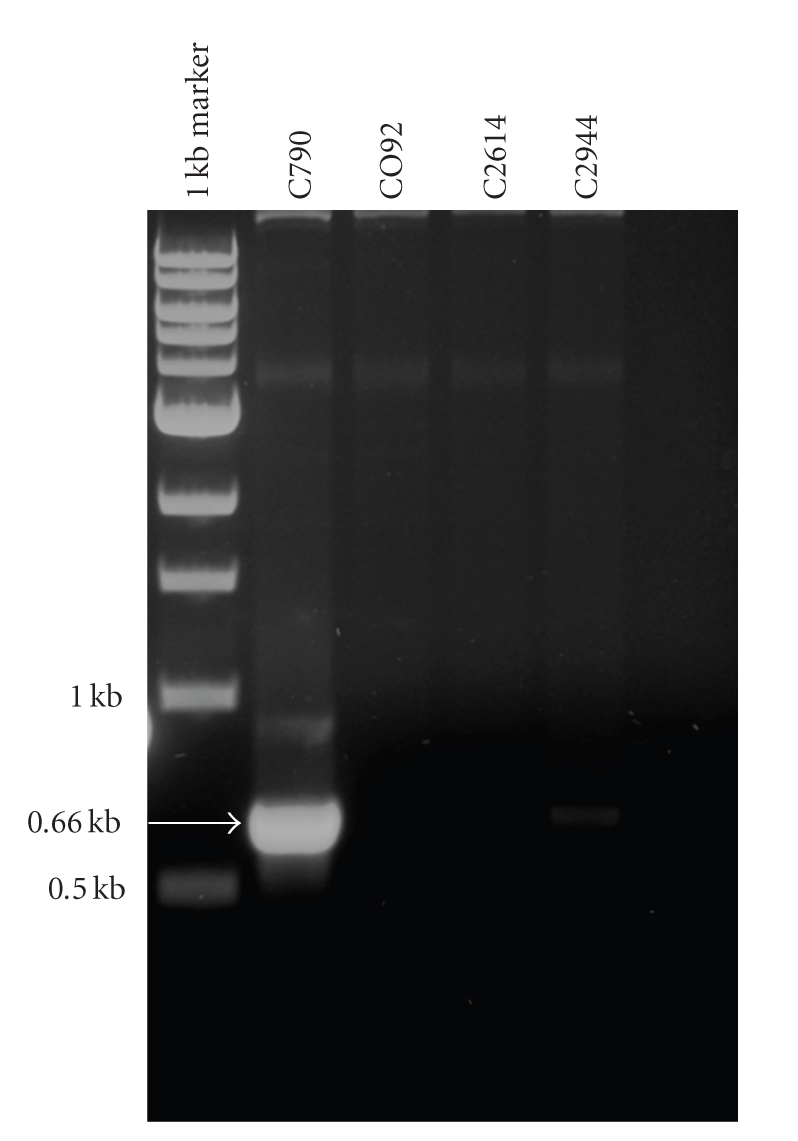
PCR amplification one of the two junctions between pMT1 and pCD1 in strain C790 which has pMT1-pCD1 chimera plasmid.

The pPCP1 plasmids in the three PCR-positive FSU strains were approximately 9.61-kb in size and had a G + C content of 45.3%, which is slightly lower than the overall G + C content (47.6%) of the KIM and CO92 strains' chromosomes [3, 4] but is similar to that of the previously characterized pPCP1 plasmid [24]. The plasmids' predicted genes encode a putative (i) transposase (1.02 kb), (ii) ATP-binding protein (782 bp) that together form the insertion sequence IS100 along with the inverted repeats, (iii) replication regulation protein (the 195 bp *rop*), (iv) transcriptional regulator (the 426 bp *pim*), (v) pesticin (the 1,074 bp *pst*), (vi) plasminogen activator (PLA) protease (the 939 bp *pla*), (vii) transcriptional regulator, and (viii and ix) two hypothetical proteins (Figure 3). Six of the genes-those (encoding the transposase, ATP-binding protein, replication regulation protein, transcriptional regulator, PLA protease, and one of the two hypothetical proteins are transcribed in the same direction, and the remaining three genes (those encoding the transcriptional regulator, pesticin, and the second hypothetical protein) are transcribed in the opposite direction (Figure 3).

 The genetic organization of the pPCP1 plasmids in the three FSU strains was similar to that of the pPCP1 plasmids in the previously characterized non-FSU strains KIM and CO92, and, to a somewhat lesser degree, strain 91001 [9, 25]. Similar to the FSU pPCP1 plasmids, the pPCP1 plasmids in strains CO92 and KIM also contain nine predicted genes the pPCP1 plasmid in strain 91001 contains ten predicted genes, consisting of the same nine genes plus one additional gene encoding a hypothetical protein; (http://www.tigr.org/). All of the FSU pPCP1 plasmids contain insertion sequence IS100, which has been found in all *Y. pestis* strains and all serotype I strains of *Y. pseudotuberculosis*, and is homologous to IS21, IS232 and IS640 [26, 27].

### 3.5. Genetic Analysis and Comparison of the pPCP1 Plasmids

We compared the nucleotide sequences of the pPCP1 plasmids in the three FSU strains to the available sequences of the pPCP1 plasmids in the four, non-FSU strains. The sequences of the pPCP1 plasmids in all of the seven strains were similar except for a few nucleotide changes, which confirm earlier observations [9, 14] about the general consistency of the size and genetic organization of the pPCP1 plasmid in *Y. pestis*. Based on the minor sequence differences we detected, five sequence types (STs) were identified among the plasmids we analyzed (Figure 3): ST1 contained three plasmids, two of which were isolated from the FSU strains C2614 and C2944, and the third plasmid from the KIM strain. ST2 had 1 plasmid from a strain isolated from China Z176003. ST3 had 1 plasmid from Krygyztan strain C790. The pPCP1 plasmids in *Y. pestis* strains CO92 and 91001 had distinct STs (ST4 and ST5, resp.). The pPCP1 plasmids in the FSU strains were found to be most genetically related (by neighbor joining tree analysis) to the pPCP1 plasmid in the KIM strain, and least related to the pPCP1 plasmid in strain 91001, which is nonpathogenic for humans [28]. The small variations in plasmid sequences were found in two intergenic regions designated variable region 1 and variable region 2 (Figure 3). Though the sequence variations are minor, variable region 1 is within promoter sequence of both pesticin and hypothetical protein as predicted by BMC promoter predicator (http://searchlauncher.bcm.tmc.edu/seq-search/gene-search.html). At this point, we do not know how this variation will affect promoter binding and transcription.

### 3.6. Analysis of *pla* Transcript Levels

The observed differences in pPCP1 copy numbers among the strains prompted us to question whether plasmid copy number correlated with expression levels of *plaA *transcript in several *Y. pestis *strains. RNA transcript levels of *plaA *were determined by RT-PCR, and expression of the chromosomal *glnA* gene was used as the reference point to calculate the relative gene expression of *pla. *The results were in general agreement with the qPCR data revealing that *pla* expression was significantly (*P* ≤ .05) higher in strain C2944 than in strain CO92. The second highest expression of *pla* was by strain C2614 (1- to 3.5-fold more than by strain CO92), followed by strain C790 (0.4- to 2.5-fold more than by strain CO92) although the differences were not statistically significant (*P* ≥ .05). Our data suggest that the three FSU strains containing the pPCP1 plasmid may produce larger amounts of PLA (and in the case of C2944, significantly larger amounts of PLA) than, the best-characterized Western North American CO92 strain.

### 3.7. *pla* and Virulence of *Y. pestis*

Although some wild-type *Y. pestis* strains lacking the pPCP1 plasmid have been reportedly retained their virulence [29, 30], pPCP1 is believed to play a major role in *Y. pestis* virulence. In addition to regulatory genes and genes encoding hypothetical proteins, the plasmid contains three genes (*pst, pim*, and *pla*) that encode proteins required for several important biochemical activities of *Y. pestis*. However, at the present time, the PLA protease, a 34.6-kDa, multifunctional plasminogen activator protease capable of degrading fibrin, coagulase, and the complement component C3 [9, 25], is the only well-documented virulence factor encoded by the pPCP1 plasmid [5]. 

The significance of *pla *in *Y. pestis* virulence has been clearly documented in literature [31, 32], and some *pla*-negative mutants have been reported to have greatly reduced virulence when administered subcutaneously [2]. Various virulence roles for the PLA protease have been proposed [9], including (i) cleaving host fibrin deposits that trap the bacterium, (ii) degrading the host's extracellular membranes, and (iii) inhibition of interleukin-8 production. More recently, *pla* expression has been reported to be essential for the development of primary pneumonic plague in virulent CO92 strain, but not in pPCP1-deficient Pestoides F, in addition to being required for the bubonic form of plague and increased potential for epidemic spread [30, 31, 33, 34]. Other studies have implicated Pla in resistance to cationic antimicrobial peptides such as cathelicidin [35]. In view of these data, our observation that some FSU strains are capable of producing significantly larger amounts of PLA than does the highly pathogenic CO92 raises interesting questions about the pathogenic potential of these strains. In this context, and to put our findings into further perspective, several reports in the “Soviet” literature suggest that *Y. pestis* virulence for various laboratory animals, including guinea pigs, varies dramatically depending on its plasmid composition and the plague focus in the Caucasus from which the strains were isolated. For example, *Y. pestis* strains isolated in the Leninakan focus (in the Shirak Highlands of Armenia) were found to be more virulent in guinea pigs than are strains obtained from the Zanzegur-Karabakh region (southeastern range of the Lesser Caucasus mountains) [36]. Similar observations have been reported for *Y. pestis* strains isolated in the Armenian Highlands [37], the Dagestan-Highland focus [38], and the Gissar and Talas regions [39]. Data comparing the plasmid compositions, the plasmid copy numbers, and *pla*-expression in those strains are not available. However, given the differences we observed in the *pla*-expression levels in various FSU strains, additional studies seem warranted to determine the impact of pPCP1 copy number and PLA production on the virulence of the FSU *Y. pestis* strains. The resulting data would help to advance understanding of the genetic composition and virulence traits of the *Y. pestis* strain population in the FSU (including FSU regions from which such data are either very scarce or not available), and they may also aid the development of advanced methods for differentiating highly virulent and less virulent strains of *Y. pestis*.

## Supplementary Material

A. Primers used for, amplifying 7 loci for MLVA, primer walking of pPCP1 plasmids, detecting pPCP1 specific genes, quantitative PCR for plasmid DNA, and RNA.
B. Each of the 7 MLVA loci were PCR amplified and the purified PCR product was sequence in both directions.
The Tandem Repeat Finder was used to determine the number of repeats.

## Figures and Tables

**Table 1:** Ratio of *pla/glnA* gene copy numbers determined by qPCR and *pla* gene expression by the Livak method.

Strains	pla/glnA	Relative *pla* gene expression
C790	15.01 ± 1.14	1.43 ± 1.03
C2614	13.21 ± 0.64	2.24 ± 1.29
C2944	18.16 ± 2.99	3.15 ± 1.31
CO92	6.48 ± 2.07	1.00
