# Supplementary material for: Characterization of pPCP1 Plasmids in Yersinia pestis Strains Isolated from the Former Soviet Union
Source: Int J Microbiol. 2010 Dec 20;2010:760819. doi: 10.1155/2010/760819 (PMC3010648; doi:10.1155/2010/760819)
Supplement: Supplementary file 1 — A. Primers used for, amplifying 7 loci for MLVA, primer walking of pPCP1 plasmids, detecting pPCP1 specific genes, quantitative PCR for plasmid DNA, and RNA. B. Each of the 7 MLVA loci were PCR amplified and the purified PCR product was sequence in both directions. The Tandem Repeat Finder was used to determine the number of repeats. [file 760819.f1.pdf]

Table 1. Primers used for the PCR and qPCR studies and for sequencing the pPCP1 plasmids (supplementary material)

Gene	Prmiers 5’→ 3’	Reference	Amplicon size (bp)
yp0120ms01 F	CTAAGCACAATTGTTATGCTGAACC	Pourcel et al 2004	
yp0120ms01 R	TACTGAATCTGCTTCATTGTTCAAA		
yp1290ms04 F	CGCTGTTGAAGTTTTAGTGTAAGAA	Pourcel et al 2004	
yp1290ms04 R	AAATGTAACCTGCCAAACGTG		
yp2769ms06 F	AATTTTGCTCCCCAAATAGCAT	Pourcel et al 2004	
yp2769ms06 R	TTTTCCCCATTAGCGAAATAAGTA		
yp2916ms07 F	ATACCGCTACGATCAGCCTCTAT	Pourcel et al 2004	
yp2916ms07 R	ATTTAATATTGATTTTGGGACTTGC		
yp1335ms46 F	CAGGTTTTACGTTATTTTCTGAAGG	Pourcel et al 2004	
yp1335ms46 R	CAGCATGAAGTATGACGGGTATATTA		
yp4280ms62 F	TTTAGTCTTGATTAAGCTGCGTTTT	Pourcel et al 2004	
yp4280ms62 R	ACGGAAGACAACCTTATTATTGATG		
yp1580ms70 F	AAACCAACGGTTCATATTGAATAAA	Pourcel et al 2004	
yp1580ms70 R	CTTCTTCCGCTATTTTCCTACAGA		
Pla F1	AAG TTC TAT TGT GGC AAC C	This study	470
Pla R1	CGTGTTTCCTGATATCCTGCT		
Pst F	GGTGTTCCGGCTTTTCTCTT	This study	201
Pst R	TTCCTGGCGGATCTTATTTG		
Pim F	CATGGCATCTATGGGAAACA	This study	197
Pim R	GCTACATATGTTGGCGGTCTT		
glnAF	AAGGCGGTTACTTCCCTGTT	This study	156
glnaAR	CGGGTAGCCACTTCGTTTT		
PlaF2	TGTGAAAGGCTGGTTACTCC	This study	145
PlaR2	CCTATTACCCGCACTCCTTT		
pcp1F	CGGTGCAATAGTGATCCACA	This study	660
pcp1R	CACGTTGCAGAACCACAGTT		
pcp2F	GGAGACCTGCCATCGTAATG	This study	673
pcp2R	GAAGCAGATGCTCCAGGAAG		
pcp3F	GTTGCAACTGGAAAGCCTTA	This study	663
pcp3R	TGAACGACATGTGAGTGGTG		
pcp4F	GGTGATGCAGCACTGACCT	This study	654
pcp4R	TCTGACGACATGAAACGAAAA		
pcp5F	GTAACGCGATTTTCCGTACC	This study	670
pcp5R	GCATCCAGAGCATCCAGTTT		
pcp6F	ATGGCGCGATTTATCAGAAG	This study	659
pcp6R	TTAAGTCCTGTCGGGTTTCG		

pcp7F	CCTGACGAGCATCACAAAAA	This study	650
pcp7R	GTGTTACCGCAGCAATACCC		
pcp8F	CCGATCAGTTCAGCAGAAGA	This study	650
pcp8R	TGAGGGGCTTGCTATTAATGAT		
pcp9F	AAAGAAATCTATCCCTGGTTGC	This study	649
pcp9F	GTTGACCACGATATTTTCTCCA		
pcp10F	CGGATTAAACCCTTCAAAACC	This study	655
pcp10F	CAGAGAAAATAAAGCGGCGTA		
pcp11F	CCATTGTATCTGACATAAAAATTCC	This study	689
pcp11F	CGTTTTCTCGTCGTCTGACA		
pcp12F	CCTGGATATTTTCCACCGTTT	This study	670
pcp12F	GAACCACCTGTAGCTGTCCAA		
pcp13F	GGCTGGTTACTCCAGGATGA	This study	686
pcp13F	TGGAGAATGTCAAGGCAAAA		
pcp14F	TGACCAACCTTCAGATGTGTG	This study	698
pcp14F	CAGGAAAAGGTTTTGGCTGA		
pcp15F	GCATATGGCCTGGGTAACTC	This study	696
pcp15F	GGGAGCGTTTTAAGCAGGTT		
pcp16F	CATGCGTATCCAGGATGAGT	This study	770
pcp16F	GCAGATTACCCCCTGGATCT		

Table 2. Tandem repeat numbers for 7 loci used in this study (supplementary material)

Strains	ms01	ms04	ms06	ms07	ms46	ms62	ms70
1390G	7.1	4.8	7.6	8.3	6.0	11.3	2.7
1391G	7.1	4.8	7.6	8.3	6.0	11.3	2.6
1392G	7.1	4.8	7.6	8.3	6.0	11.3	2.7
1393G	7.1	4.8	7.6	8.3	6.0	11.3	2.7
1412G	7.6	4.8	7.4	8.3	6.0	11.3	2.1
1413G	7.3	4.8	7.6	8.3	6.0	11.3	2.6
1670G	7.3	4.8	7.6	8.3	6.0	11.3	2.6
1851G	7.1	4.8	7.6	8.3	6.0	11.3	2.7
1852G	7.1	4.8	7.5	8.2	6.0	11.3	2.6
1853G	7.3	4.8	7.6	8.3	6.0	11.3	3.5
1952G	7.1	4.8	6.7	8.3	6.0	11.3	2.6
1953G	7.1	4.8	7.6	8.3	6.0	11.3	2.7
1954G	7.1	4.8	7.3	8.3	6.0	11.3	2.7
2095G	7.1	4.8	7.6	8.3	6.0	11.3	2.7
3064G	7.1	4.8	7.6	8.3	6.0	11.3	2.7
3065G	7.1	4.8	7.3	8.3	6.0	11.3	2.7
3066G	7.1	4.8	7.6	8.3	6.0	11.3	2.6
3067G	7.1	4.8	7.6	8.3	6.0	11.3	2.1
3072G	7.3	4.8	7.6	8.3	6.0	11.3	2.6
3073G	7.1	4.8	7.6	8.3	6.0	11.3	2.7
3082G	7.1	4.8	7.6	8.3	6.0	11.3	2.7
3083G	7.1	4.8	7.6	8.3	6.0	11.3	2.6
3757G	7.1	4.8	7.6	8.2	6.0	9.3	2.7
3758G	7.1	4.8	7.3	8.3	6.0	11.3	2.1
3768G	7.1	4.8	7.6	8.3	6.0	11.3	2.7
3769G	7.1	4.8	7.6	8.3	6.0	11.3	2.6
3770G	7.1	4.8	7.6	8.3	6.0	11.3	2.3
8786G	7.1	4.8	7.6	6.4	6.0	11.3	2.6
8787G	7.1	4.8	7.6	7.4	6.0	11.3	2.7
8788G	7.1	4.8	7.6	8.3	6.0	11.3	3.0
8789G	7.3	4.8	7.6	7.4	6.0	11.3	2.7
8790G	7.1	4.8	7.6	8.3	6.0	11.3	2.7
8791G	7.1	4.8	7.6	8.3	6.0	11.3	2.7
8792G	7.1	4.8	7.6	8.3	6.0	11.3	2.1
8793G	7.2	4.8	7.6	8.3	6.0	11.3	2.6
8794G	7.1	4.8	7.5	8.3	6.0	11.3	2.7
8906G	7.1	4.8	7.6	8.3	6.0	11.3	2.6
8907G	7.3	4.8	6.6	8.3	6.0	11.3	2.6
8908G	7.1	4.8	7.6	8.3	6.0	11.3	2.7
C1045	7.1	5.5	3.5	7.4	0.0	13.3	4.7
C14735	7.3	4.8	7.5	9.4	6.0	10.3	2.7
C1522	7.3	4.8	7.5	9.4	6.0	10.3	7.6
C2614	7.1	5.8	3.5	8.3	0.0	17.3	7.7
C2944	7.1	5.8	3.5	8.3	0.0	17.3	6.6
C771	7.1	5.8	3.5	8.2	3.4	13.3	6.7
C790	6.1	6.8	7.5	7.4	0.0	7.3	2.1
CO92	8.3	7.8	8.5	9.3	7.0	6.3	5.7
KIM	6.1	5.8	3.5	6.4	0.0	10.3	5.7
